# Two-dimensional speckle tracking for the assessment of coronary artery disease during dobutamine stress echo: clinical tool or merely research method

**DOI:** 10.1186/s12947-015-0038-z

**Published:** 2015-10-24

**Authors:** Constantina Aggeli, Stauroula Lagoudakou, Ioannis Felekos, Vasiliki Panagopoulou, Stellios Kastellanos, Konstantinos Toutouzas, George Roussakis, Dimitris Tousoulis

**Affiliations:** 1st Cardiology Department, Hippokration Hospital, Athens Medical School, 114 Vas. Sophias Ave, Athens, Greece

**Keywords:** Speckle tracking, Coronary artery disease, Stress echo

## Abstract

**Background:**

Two-dimensional speckle tracking provides valuable information for regional wall motion abnormalities. The purpose of this study was to determine the diagnostic value of left ventricular longitudinal strain and torsion to diagnose coronary artery disease during dobutamine stress echocardiography.

**Methods:**

We studied 100 patients (mean age 60.8 ± 10.7 years, 72 male) with known or suspected coronary artery disease, excluding those with prior history of transmural infraction. All of them underwent dobutamine stress echo and coronary angiography within one month. Wall-motion score index, left ventricular global longitudinal strain and torsion were measured at rest and peak stress. Additionally, the respective differences between rest and stress were also calculated. Optimal cut-offs were derived from receiver operating characteristic curves for strain and torsion values.

**Results:**

Mean left ventricular ejection fraction was 55 ± 5.4 %. Coronary angiography revealed significant lesions in 67 patients. Values regarding sensitivity, and specificity for wall motion score index difference were 78 % and 88 % respectively (area under curve 0.84). Global longitudinal strain difference (median 0.5 %) illustrated 81 % sensitivity and 72 % specificity for disease detection (area under curve 0.80, cut-off value ≤0 %). The respective values for torsion difference (median 4.7°) were 81 % and 82 % (area under curve 0.76, cut-off value ≤6.5°). Combination of wall motion score index difference and torsion difference for disease detection showed 91 % sensitivity and 79 % specificity (area under curve 0.85).

**Conclusions:**

The implementation of speckle tracking during dobutamine stress echo could serve as an adjunct method for coronary artery disease assessment, providing quantitative diagnostic information.

## Background

Timely and prompt detection of coronary artery disease (CAD) is of paramount importance for patient management in daily clinical practice. Dobutamine stress echocardiography (DSE) is a non-invasive method with established diagnostic accuracy. Still, it remains subjective, mainly because of its dependence from operator experience on image acquisition and interpretation [1, 2].

Speckle tracking echocardiography is a novel method for the assessment of global and regional left ventricular myocardial function. It provides valuable insights to myocardial deformation by quantifying strain and torsion with the inherent advantage of being angle-independent [3, 4]. From the clinical perspective, it has already been used for assessing different myocardial and systemic diseases, where it can predict probable sub-clinical cardiac disorder earlier in the course of the disease process when conventional 2D echocardiography appears to be normal [5–9].

Speckle tracking derivatives are less susceptible to translational motion and tethering, which lead to erroneous qualitative evaluation of stress echo images. So far few studies have been published exploring the implementation and clinical utility of speckle tracking imaging during DSE [10–13]. The aim of the current study was to assess the feasibility of speckle tracking during DSE and evaluate the diagnostic accuracy of 2D global longitudinal strain and torsion, along with its additive role over wall motion abnormalities interpretation for the diagnosis of CAD.

## Methods

### Study population

We studied *114* consecutive patients with known or suspected coronary artery disease who were referred to our tertiary center for evaluation of myocardial ischemia or coronary angiography. Fourteen patients were rejected due to poor acoustic window, unsuitable for speckle tracking echo. Eventually 100 patients were included in the study. All of them underwent DSE and coronary angiography within one month. Patients with depressed left ventricular function (EF < 45 % at rest), significant valvular heart disease, left bundle branch block on ECG, pacemaker and history of coronary artery bypass surgery were also excluded. The study protocol was approved by the ethics committee of the Hippokration Hospital of Athens and all patients gave written informed consent.

### Image acquisition

All studies were performed with an iE33 ultrasound machine (Philips Medical Systems, USA), equipped with the 2.5 MHz S5-1 transducer. Images were obtained with patients being positioned in the left lateral decubitus position, in the parasternal short axis (mitral leaflet, annular and apex level) and apical 4-, 3- and 2-chamber views at rest and peak dobutamine infusion. Two-dimensional grayscale images were obtained at a frame rate of 70–80 Hz in order to be optimal for speckle tracking, during three cardiac cycles and were then digitally stored for off-line analysis. Quantitative measurements of longitudinal strain and torsion were made using the QLAB 9.0 software package.

### Dobutamine stress echo

Dobutamine stress echo was performed using a standard protocol [14]. An intravenous line was placed and dobutamine was infused in four 3-min stages: 10-20-30-40 μg/kg/min. Atropine was given at the end of 12-min period, as required (at a dose of 0.2-1 mg) to achieve the age-adjusted target heart rate [0.9 x (220-age)], unless contraindicated. A short-acting beta-blocker (esmolol 20–40 mg) was administered upon test completion to all patients (except those with a known history of severe bronchospasm) in order to accelerate normalization of the heart rate. All patients were asked to withdraw b-blockers for at least 48 h prior testing.

The stress protocol was terminated if : (1) the age-adjusted target heart rate was achieved; (2) four or more contiguous segments showed signs of ischemia; (3) the patient reported intense chest pain; (4) the electrocardiogram showed VT, ventricular bigeminy or trigeminy, or multiform premature ventricular complexes; (5) if any other grade > 2 adverse event appeared.

Wall motion was assessed by an expert observer. The 17-segment model of the left ventricle was used [15]. Myocardial segments were graded according to their wall motion at baseline and at each stage of the protocol, as normokinetic (grade 0), hypokinetic (grade 1), akinetic (grade 2) or dyskinetic (grade 3). Any deterioration by one grade or more, from baseline to peak stress, in two or more contiguous segments was considered indicative of ischemia. Wall motion score index (WMSI) was calculated at rest and peak stress and then the difference (value at stress minus value at rest, WMSIΔ) was derived.

### Speckle tracking echocardiography

**Fig. 1:**
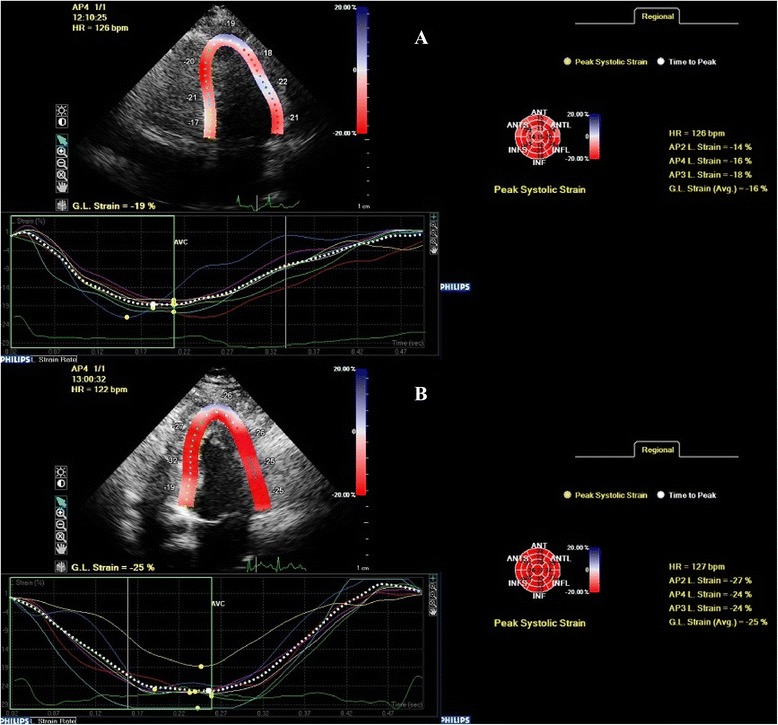
A patient with LAD disease (**a**) and a normal patient (**b**) are illustrated (apical 4-chamber view and bull’s-eye) at peak stress

Longitudinal strain and torsion measurements with the implementation of 2D speckle tracking were performed as previously described [5]. End-systole has been identified as corresponding to the aortic valve closure measured by pulsed-Doppler. The operator traced the endocardial border on an end-diastolic frame and the software automatically tracked the border on the subsequent frames. Adequate tracking can then be verified in real-time and corrected if deemed necessary by adjusting the region of interest or manually correcting the border to ensure optimal tracking. After the tracking process is completed myocardial deformation is plotted in time versus strain graphs, where it is possible to identify the different phases of cardiac cycle (Fig. 1).

To assess left ventricle rotation, six tracking points were placed on the myocardium, avoiding the pericardium on an end-diastolic frame in each parasternal short-axis image as determined automatically by the software algorithm. Counterclockwise rotation, as viewed from the apex, was expressed as a positive value; clockwise rotation was expressed as a negative value. Analysis was performed to evaluate the peak apical rotation during the ejection phase, the basal rotation at a time isochronous with that of peak apical rotation during the ejection phase and the net instantaneous torsion of the left ventricle, which was calculated as peak apical minus the basal rotation at a time isochronous with peak apical rotation.

Anterior and posterior circulation was evaluated separately as proposed by Geleijnse et al. [14]. For anterior circulation the mean value of strain was calculated using all the segments which are supplied from the LAD, which are the anterior, anteroseptal, apical and midseptal segments. Similarly for posterior circulation the mean value of strain was calculated of all segments which are supplied from the RCA and LCX taken together, which are the basal, and mid inferior, basal inferoseptum, basal and mid lateral and basal and mid posterior. Finally the change (Δ-delta) in longitudinal strain and torsion (value at peak stress minus value at rest) was estimated.

### Coronary angiography

All patients underwent coronary angiography within one month according to the Judkins technique. All images were evaluated by an experienced operator, and significant coronary artery disease was defined as >70 % luminar diameter stenosis.

### Statistical analysis

Data analysis was performed using the SPSS 20.0 statistical package for Windows (SPSS Inc., Chicago). Continuous variables were presented as mean ± SD, whereas categorical variables were expressed as percentages. The chi-square test was used to compare categorical variables. Independent t-tests were used for comparison of matched segments in patients with and without CAD. Receiver operating characteristics curves (ROC) for each of the strain and torsion parameters was calculated and the optimal stain and torsion cutoff values for predicting CAD were then determined. In all cases, a *p*-value < 0.05, was regarded as statistically significant. For global intra- and interobserver variability, 10 patients were selected at random, and measurements were repeated by the same observer on the same echocardiographic images 2 weeks apart and by another independent observer. The inter-observer variability was assessed using a two-way mixed, absolute agreement, single measures intra-class correlation.

## Results

Patient demographic data and hemodynamics are illustrated in detail in Tables 1 and 2. Mean age was 60.8 ± 10.7 years old (72 male), with mean left ventricular EF 55 ± 5.5 %. Coronary angiography revealed significant CAD in 67 patients. Of them, 46 had single-vessel disease while the rest of the study population had multi-vessel disease.

**Table 1:** Patient demographics

Baseline characteristics	Total (*n* = 100)	CAD(+) (*n* = 67)	CAD (−) (*n* = 33)	*p*-value
Male, *n* (%)	72 (72 %)	54 (75 %)	18 (25 %)	0.006
Age (year)	60.8 ± 10.7	63.2 ± 10.3	55.8 ± 9.9	0.001
Hypertension, *n* (%)	59 (59 %)	44 (65.7 %)	15(45.5 %)	0.05
Diabetes, *n* (%)	23 (23 %)	21 (31.3 %)	2 (6.1 %)	0.05
Dyslipidaemia, *n* (%)	69 (69 %)	52 (77.6 %)	17 (51.5 %)	0.08
Smoking, *n* (%)	30 (30 %)	21(31.3 %)	9 (27.3 %)	0.7
Family Hx for CAD, *n* (%)	31 (31 %)	18 (26.9 %)	13 (39.4 %)	0.2
LV ejection fraction, (%)	55 ± 5.4	53.4 ± 5.3	58.2 ± 4.1	0.6
WMSI rest	1.06 ± 0.99	1.08 ± 0.1	1.02 ± 0.5	<0.05
WMSI stress	1.20 ± 0.2	1.28 ± 0.18	1.02 ± 0.07	<0.001
WMSIΔ	0.13 ± 0.19	0.2 ± 0.19	0.003 ± 0.08	<0.001
Coronary artery disease, *n* (%)	67 (67 %)			
LAD	38 (38 %)			
LCX	29 (29 %)			
RCA	28 (28 %)			
1-vessel disease	46 (46 %)			
2-vessel disease	14 (14 %)			
3-vessel disease	7 (7 %)

**Table 2:** Patient hemodynamics at rest and stress

Hemodynamics	Rest	Stress
Systolic BP (mmHg)	138 ± 15	167 ± 10
Diastolic BP (mmHg)	82 ± 8	89 ± 6
HR (bpm)	73 ± 7	135 ± 12

We analyzed 3400 segments both in rest and stress. A total of 425 of the analyzed myocardial segments were rejected due to poor tracking. More specific, in rest the feasibility rates were 90 % (170 segments could not be analyzed), whereas in peak stress the feasibility rates were 85 % (255 segments could not be analyzed). In addition, we sought to determine inter and intra-observer variability at rest and peak stress. The correlation coefficient for the same observer at rest was 0.95 for 2D strain and 0.91 for torsion, while between the 2 observers were 0.91 and 0.89 respectively. At peak stress, the respective values for a single observer were 0.87 for GLS and 0.82 for torsion, while between two observes were 0.83 and 0.80.

**Fig. 2:**
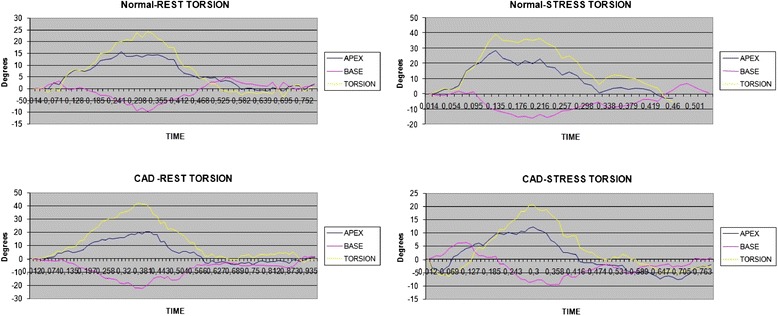
Torsion curves, along with apical and base rotations are depicted at rest and peak stress in a normal patient and a patient with CAD

**Fig. 3:**
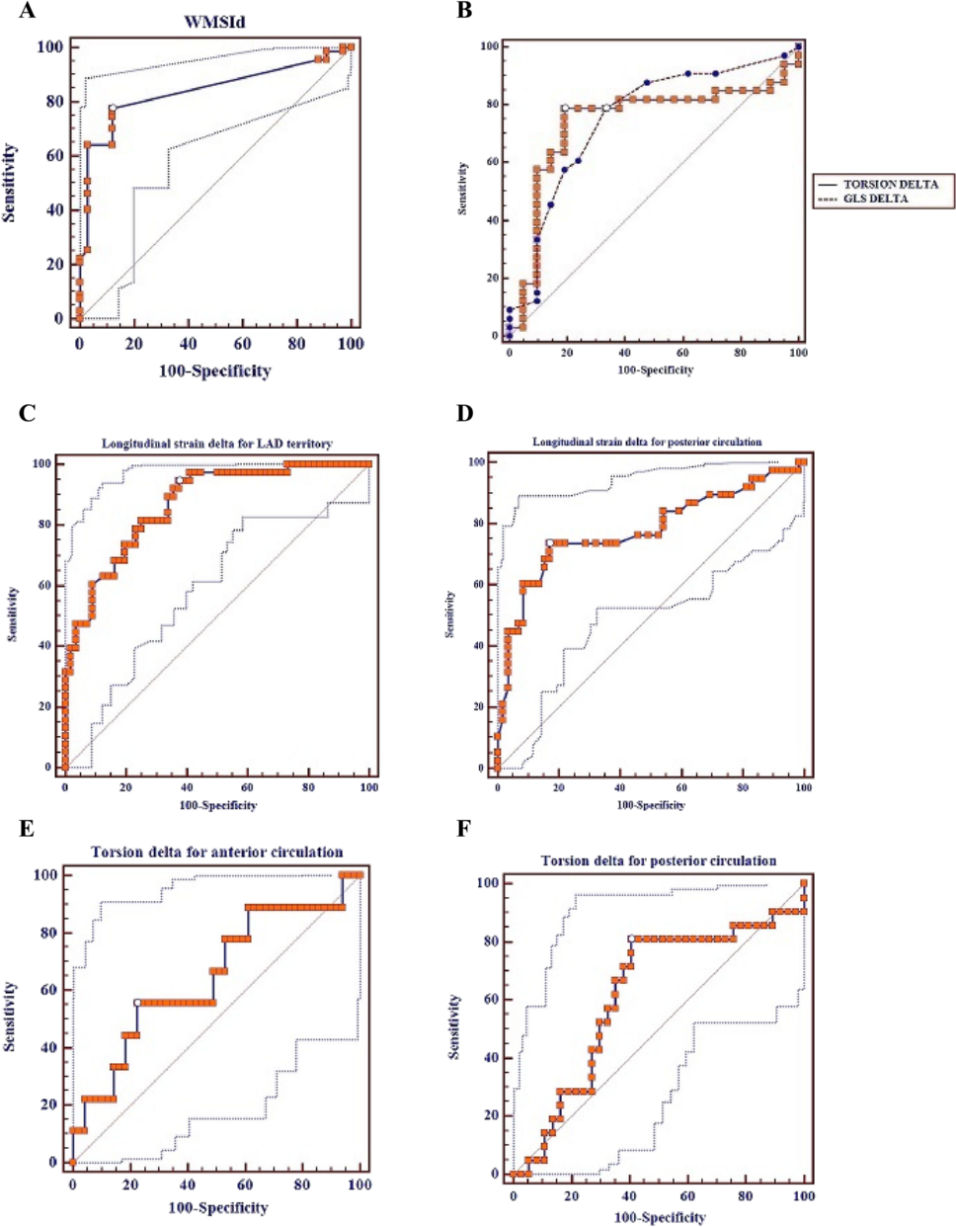
Roc Curves regarding (**a**) wall-motion score index delta (WMSId), (**b**) Global Longitudinal strain (GLS) delta and Torsion delta, (**c**) Global Longitudinal strain delta for LAD and (**d**) posterior circulation disease and (**e**) Torsion delta for anterior and (**f**) posterior circulation disease

Wall-motion score index at rest was 1.06 ± 0.99, while at stress was 1.20 ± 0.20. The difference in values of WMSI between stress and rest (WMSIΔ), exhibited 78 % sensitivity, 88 % specificity (AUC 0.84, cut-off value >0). Speckle tracking echo measurements are shown in Table 3. In specific, reduced GLSΔ values were reported in patients with CAD as compared to those without (−2.23 ± 3.36 % vs. 1.56 ± 3.15 % respectively, *p* < 0.005). Similarly, torsionΔ values were significantly reduced in ischemic patients as compared to normal persons (−2.42 ± 13.96° vs.9.50 ± 10.46° respectively, *p* < 0.005) (Fig. 2). Global longitudinal strain delta (Δ) illustrated 81 % sensitivity and 72 % specificity for the detection of CAD (AUC 0.80, cut-off value ≤0 %). The respective values for torsion delta (Δ) were 81 and 82 % (AUC 0.76, cut-off value ≤6.46°) (Fig. 3).

**Table 3:** Speckle tracking measurements

	CAD (+)	CAD (−)	*P*-value
Global longitudinal peak systolic strain (%)			
Rest	−21.16 ± 3.32	−21.59 ± 2.33	0.46
Peak dose	−18.95 ± 4.34	−23.16 ± 3.30	<0.001
Delta	−2.23 ± 3.36	1.56 ± 3.15	<0.001
Global torsion (^o^)			
Rest	21.77 ± 5.47	21.27 ± 7.61	0.790
Peak dose	19.65 ± 13.57	30.76 ± 12.03	0.002
Delta	−2.42 ± 13.96	9.50 ± 10.46	0.001
Base rotation (^o^)			
Rest	−9.28 ± 2.87	−9.74 ± 2.98	0.560
Peak dose	−8.76 ± 5.28	−12.60 ± 4.63	0.006
Delta	−0.52 ± 6.24	2.84 ± 3.61	0.012
Apex rotation (^o^)			
Rest	14.03 ± 4.02	14.57 ± 5.62	0.692
Peak dose	13.13 ± 9.29	21.39 ± 8.84	0.001
Delta	−0.90 ± 8.88	6.76 ± 8.69	0.002

The combination of GLSΔ and ΤorsionΔ for the detection of CAD showed 97 % sensitivity and 52 % specificity (AUC 0.77). The diagnostic value of the combined GLSΔ and ΤorsionΔ assessment was more sensitive as compared to wall-motion assessment alone (*p* < 0.05). The combined assessment of GLSΔ and WMSIΔ revealed 87 % sensitivity and 73 % specificity (AUC 0.80), not statistically significant different than either indice alone. On the other hand the combined assessment of TorsionΔ and WMSIΔ revealed 91 % sensitivity and 79 % specificity (AUC 0.85), higher than either indice separately (without the differences being statistically significant though). Comparison of AUC is depicted in detail in Table 4.

**Table 4:** Comparison of AUC

	AUC	SE	95 % CI
GLSΔ	0,80	0,0711	0,615 to 0,859
ΤorsionΔ	0,76	0,0739	0,601 to 0,849
WMSIΔ	0,84	0,0618	0,668 to 0,896
Torsion and GLS combined	0,77	0,0737	0,619 to 0,862
GLSΔ vs WMSIΔ			
Significance level		*P* = 0,57	
ΤorsionΔ vs WMSIΔ			
Significance level		*P* = 0,51	
WMSIΔ vs Torsion and GLS combined			
Significance level		*P* = 0,59	

In addition, we reclassified our analysis according to coronary vessel territory. Longitudinal strain parameters are depicted in Table 5. It is shown that longitudinal strain delta(Δ) values regarding patients with LAD disease are reduced in comparison to those with normal LAD (−5.46 ± 5.27 % vs. 1.76 ± 3.77 %, *p* < 0.001). Again, for posterior circulation strain values were changed in similar fashion (−2.83 ± 5.11 % vs. 2.18 ± 3.95 %, *p* < 0.001). Area under the curve for LAD detection was 0.87, reporting 94.7 % sensitivity and 62.5 % specificity (cut-off value ≤1.67 %). For posterior circulation AUC was 0.78 (74 % sensitivity, 83 % specificity, cut-off value ≤ −2 %). Changes in torsion values between rest and stress were able to predict LAD disease with 56 % sensitivity and 78 % specificity (AUC 0.65, cut-off value ≤ 10.1°), while for posterior circulation the reported values were 81 and 60 % respectively (AUC 0.62, cut-off value ≤ 5.9°) (Fig. 3).

**Table 5:** GLS (%) parameters per vessel

	LAD disease (+)	LAD disease (−)	*p*-value
Rest	−21.86 ± 3.09	−23.20 ± 2.87	0.03
Peak dose	−16.38 ± 5.76	−24.96 ± 4.42	<0.001
Delta	−5.46 ± 5.27	1.76 ± 3.77	<0.001
	LCX disease (+)	LCX disease (−)	
Rest	−19.80 ± 3.16	−21.70 ± 2.88	0.01
Peak dose	−16.13 ± 4.53	−23.43 ± 3.69	<0.001
Delta	−3.59 ± 4.91	1.51 ± 4.14	<0.001
	RCA disease (+)	RCA disease (−)	
Rest	−18.73 ± 3.052	−20.23 ± 2.87	0.04
Peak dose	−15.69 ± 5.96	−22.11 ± 4.21	<0.001
Delta	−3.47 ± 5.22	1.65 ± 4.17	<0.001
	LCX-RCA disease (+)	LCX-RCA disease (−)	
Rest	−19.28 ± 2.10	−20.29 ± 2.33	0.03
Peak dose	−16.43 ± 5.30	−22.42 ± 3.95	<0.001
Delta	−2.83 ± 5.11	2.18 ± 3.95	<0.001

## Discussion

The findings of our study demonstrate that the measurement of both 2D-longitudinal peak systolic strain and LV torsion during dobutamine stress echocardiography is feasible and provide quantifiable information for the identification of myocardial ischemia in patients without previous MI, with good overall reproducibility. It is now well established that speckle tracking is feasible during DSE. Our feasibility and reproducibility rates match those reported by other authors [16, 17]. Initial studies have shown that two dimensional (2D) strain measurements can reveal wall motion abnormalities even at rest in high risk patients [10–12].

We found that the regional strain for anterior and posterior circulation revealed statistically significant difference between the two groups at rest. According to Choi et al., patients with left main or three-vessel CAD without regional wall motion abnormalities have significantly lower peak systolic longitudinal strain at rest, reflecting the early impairment of longitudinal strain in the ischemic cascade [10]. Furthermore, 2D strain can be calculated at any stage of DSE giving significant information for the presence of ischemia, even at low dose stage [18–20]. Recently, it was shown that the detection of severe CAD (three-vessel disease) can been accomplished during the early stages of DSE before the occurrence of regional wall motion abnormalities, as the longitudinal strain is lower in patients with CAD [21].

Another study has revealed that longitudinal strain had higher diagnostic accuracy than circumferential and radial strains and reported similar diagnostic accuracy as expert wall motion analysis for the detection of CAD [22]. Considering this, we used only the longitudinal strain as the best parameter to detect ischemia. Moreover ischemic changes primarily affect long-axis function, as the subendocardial myocardial fibers, which are mainly longitudinal oriented, are more susceptible to ischemia.

We demonstrated that the combined assessment of GLSΔ and WMSIΔ, as well as Torsion Δ and WMSIΔ, failed to show better diagnostic ability during DSE. Perhaps, this could attributed to the lower feasibility rates at peak stress, 255 segments could not be analyzed. Still, it offers enhanced sensitivity than each indice alone, on the expense however of lower specificity, especially in the combination of GLSΔ and WMISΔ. The enhanced sensitivity could prove valuable in clinical practice when assessing intermediate to high risk patients, in order to minimize false negative rates, which could have a negative impact on their outcomes. Similar results have been highlighted by Arnold et al., who found that the combination of GLS and WMSI offers enhanced diagnostic ability [22]. We took this information one step further by exploring the additive value of torsion.

The assessment of the LV torsion by speckle tracking echocardiography is feasible, as initial studies have shown [23–25], but very few of them have incorporated the LV torsion measurement during DSE [26, 27]. Torsion can be a marker of the global systolic myocardial performance, directly related to fiber architecture and the contractility of the myocardium. In our study, we noticed reduction of the LV torsion at peak stress in patients with CAD. On the contrary, patients without CAD had a significant augmentation of the LV torsion, which is evidence of the increased contractility of the LV at dobutamine peak dose. Interestingly, LV torsion was more sensitive in detecting posterior circulation disease, perhaps due to the fact that the apical rotation is less pronounced than basal rotation. This was also previously shown by Knudtson et al. [28], who demonstrated reduction in apical LV rotation with balloon occlusion of the LAD. Similar findings of impairment in LV apical rotation and torsion in response to coronary occlusion have been reported in the anaesthetized dogs [29].

In addition, we analyzed the change in the regional strain according to the coronary arteries’ supply territory and could assess anterior or posterior circulation involvement separately. In our study, 2D-strain exhibited superior diagnostic efficiency for the evaluation of anterior coronary circulation, as compared to posterior circulation. Our results seem to verify those reported by Hanekom et al. [13]. The lower diagnostic reported measures in the posterior circulation could be attributed to tracking problems in the posterolateral segments. Moreover, the same group compared the accuracy of the speckle tracking longitudinal strain and the accuracy of the tissue velocity imaging (TVI)-based strain during DSE and showed that they were similar in the anterior, but not in the posterior circulation.

### Limitations

It should be noted though, that there is probable selection bias with regards to participants’ BMI. Obese people were rejected from our study, which in turn could lead to feasibility overestimation. Patients with transmural myocardial infarction have been excluded from this study due to abnormal torsion at rest, which could potentially result into different torsion response during stress echo. Moreover, the aim of the study was to investigate the diagnostic accuracies of strain analyses for detection of significant CAD on angiography and not the assessment of myocardial viabilities and therefore patients with severely depressed resting LV ejection fraction (LVEF ≤40 %) were excluded. Finally, there are certain limitations inherent to the technique of STE, which could reduce the signal-to-noise ratio especially during higher heart rates. Another limitation is that we did not use contrast agents to improve wall motion interpretation. Although this might have increased the diagnostic capability of WMSI, it would also have made speckle tracking imaging impossible.

### Clinical perspective

Given that deformation parameters and specifically global longitudinal strain can detect ischemia in an early stage, it could potentially provide better risk stratification for our patients. Furthermore, LV torsion was more sensitive in detecting posterior circulation disease, perhaps due to the fact that the apical rotation is less pronounced than basal rotation. This finding could be translated into clinical efficacy, since the ability of DSE to diagnose posterior circulation disease is somehow obscured by imaging difficulties of this specific region of interest. In addition, novel software algorithms allow for easier, faster and reproducible measurements, transforming speckle tracking into a robust clinical tool in the near future.

## Conclusion

In the present study we showed that the measurement of LV torsion and 2D-strain is feasible during DSE. Therefore, both of them might be used as a quantitative tool in combination with wall motion abnormalities for the detection of CAD. In addition, 2D-strain can give quantitative information for segmental ischemia, which comes in accordance with the angiographic findings.

## Abbreviations

CAD: Coronary Artery Disease
DSE: Dobutamine Stress Echocardiography
WMSI: Wall-motion score index
GLS: Global Longitudinal Strain
2D: Two Dimensional
EF: Ejection Fraction

## References

[1] Hoffmann R, Lethen H, Marwick T, Rambaldi R, Fioretti P, Pingitore A (1998). Standardized guidelines for the interpretation of dobutamine echocardiography reduce interinstitutional variance in interpretation. Am J Cardiol.

[2] Hoffmann R, Lethen H, Marwick T, Arnese M, Fioretti P, Pingitore A (1996). Analysis of interinstitutional observer agreement in interpretation of dobutamine stress echocardiograms. J Am CollCardiol.

[3] Huang SJ, Orde S (2013). From speckle tracking echocardiography to torsion: research tool today, clinical practice tomorrow. CurrOpinCrit Care.

[4] Nesbitt GC, Mankad S, Oh JK (2009). Strain imaging in echocardiography: methods and clinical applications. Int J Cardiovasc Imaging.

[5] Aggeli C, Felekos I, Tousoulis D, Gialafos E, Rapti A, Stefanadis C (2013). Myocardial mechanics for the early detection of cardiac sarcoidosis. Int J Cardiol.

[6] Marwick TH, Leano RL, Brown J, Sun JP, Hoffmann R, Lysyansky P (2009). Myocardial strain measurement with 2-dimentional speckle-tracking echocardiography: definition of normal range. JACC Cardiovasc Imaging.

[7] Xie MX, Zhang L, Lü Q, Wang XF, Han W, Zhang J (2008). Left ventricular rotation and twist in patients with hypertrophic cardiomyopathy evaluated by two-dimensional ultrasound speckle-tracking imaging. Zhongguo Yi XueKeXue Yuan XueBao.

[8] Popović ZB, Kwon DH, Mishra M, Buakhamsri A, Greenberg NL, Thamilarasan M (2008). Association between regional ventricular function and myocardial fibrosis in hypertrophic cardiomyopathy assessed by speckle tracking echocardiography and delayed hyper enhancement magnetic resonance imaging. J Am SocEchocardiogr.

[9] Lim P, Mitchell-Heggs L, Buakhamsri A, Thomas JD, Grimm RA (2009). Impact of left ventricular size on tissue Doppler and longitudinal strain by speckle tracking for assessing wall motion and mechanical dyssynchrony in candidates for cardiac resynchronization therapy. J Am SocEchocardiogr.

[10] Choi JO, Cho SW, Song YB, Cho SJ, Song BG, Lee SC (2009). Longidutinal 2D strain at rest predicts the presence of left main and three vessel coronary artery disease in patients without regional wall motion abnormality. Eur J Echocardiogr.

[11] Yang B, Daimon M, Ishii K, Kawata T, Miyazaki S, Hirose K (2013). Prediction of coronary artery stenosis at rest in patients with normal left ventricular wall motion segmental analyses using strain imaging diastolic index. Int Heart J.

[12] Montgomery DE, Puthumana JJ, Fox JM, Ogunyankin KO (2012). Global longitudinal strain aids the detection of non-obstructive coronary artery disease in the resting echocardiogram. Eur Heart J Cardiovasc Imaging.

[13] Hanekom L, Cho GY, Leano R, Jeffriess L, Marwick TH (2007). Comparison of two-dimentional speckle and tissue Doppler strain measurement during dobutamine stress echocardiography: an angiographic correlation. Eur Heart J.

[14] Geleijnse ML, Fioretti PM, Roelandt JR (1997). Methodology, feasibility, safety and diagnostic accuracy of dobutamine stress echocardiography. J Am CollCardiol.

[15] Cerqueira MD, Weissman NJ, Dilsizian V, Jacobs AK, Kaul S, Laskey WK (2002). Standardized myocardial segmentation and nomenclature for tomographic imaging of the heart: a statement for healthcare professionals from the cardiac imaging committee of the council on clinical cardiology of the american heart association. Circulation.

[16] Ryo K, Tanaka H, Kaneko A, Fukuda Y, Onishi T, Kawai H (2012). Efficacy of longitudinal speckle tracking strain in conjunction with isometric handgrip stress test for detection of ischemic myocardial segments. Echocardiography.

[17] Joyce E, Hoogslag GE, Al Amri I, Debonnaire P, Katsanos S, Bax JJ, et al. Quantitative Dobutamine Stress Echocardiography Using Speckle-Tracking Analysis versus Conventional Visual Analysis for Detection of Significant Coronary Artery Disease after ST-Segment Elevation Myocardial Infarction. J Am Soc Echocardiogr. 2015. S0894-7317(15)00548-9. 10.1016/j.echo.2015.07.023 26307373

[18] Wierzbowska-Drabik K, Hamala P, Roszczyk N, Lipiec P, Plewka M, Krecki R (2014). Feasibility and correlation of standard 2D speckle tracking echocardiography and automated function imaging derived parameters of left ventricular function during dobutamine stress test. Int J Cardiovasc Imaging.

[19] Govind SC, Gopal AS, Netyo A, Nowak J, Brodin LA, Patrianakos A (2009). Quantification of low-dose dobutamine stress using speckle tracking echocardiography in coronary artery disease. Eur J of Ecdhocardiogr.

[20] Yu Y, Villarraga HR, Saleh HK, Cha SS, Pellikka PA (2013). Can ischemia and dyssynchrony be detected during early stages of dobutamine stress echocardiography by 2-dimensional speckle tracking echocardiography?. Int J Cardiovasc Imaging.

[21] Kim HK, Sohn DW, Lee SE, Choi SY, Park JS, Kim YJ (2007). Assessment of left ventricular rotation and torsion with two-dimensional speckle tracking echocardiography. J Am SocEchocardiogr.

[22] Nq AC, Sitges M, Pham PN, da Tran T, Delgado V, Bertini M (2009). Incremental value of 2-dimentional speckle tracking strain imaging to wall motion analysis for detection of coronary artery disease in patients undergoing dobutamine stress echocardiography. Am Heart J.

[23] Notomi Y, Lysyansky P, Setser RM, Shiota T, Popović ZB, Martin-Miklovic MG (2005). Measurement of ventricular torsion by two-dimensional ultrasound speckle tracking imaging. J Am CollCardiol.

[24] Phillips AA, Cote AT, Bredin SS, Warburton DE (2012). Heart disease and left ventricular rotation – a systematic review and quantitative summary. BMC Cardiovasc Disord.

[25] Joyce E, Leong DP, Hoogslag GE, van Herck PL, Debonnaire P, Abate E (2014). Left ventricular twist during dobutamine stress echocardiography after acute myocardial infarction: association with reverse remodeling. Int J Cardiovasc Imaging.

[26] Bansal M, Leano RL, Marwick TH (2008). Clinical assessment of left ventricular systolic torsion: effects of myocardial infarction and ischemia. J Am SocEchocardiogr.

[27] Knudtson ML, Galbraith PD, Hildebrand KL (1997). Dynamics of left ventricular apex rotation during angioplasty: a sensitive index of ischemic dysfunction. Circulation.

[28] Helle-Valle T, Crosby J, Edvardsen T (2005). New noninvasive method for assessment of left ventricular rotation: speckle tracking echocardiography. Circulation.

[29] Park SM, Hong SJ, Ahn CM, Kim YH, Kim JS, Park JH (2012). Different impacts of acute myocardial infarction on left ventricular apical and basal rotation. Eur Heart J Cardiovasc Imaging.

